# Relaxed Molecular Clock Provides Evidence for Long-Distance Dispersal of *Nothofagus* (Southern Beech)

**DOI:** 10.1371/journal.pbio.0030014

**Published:** 2005-01-04

**Authors:** Michael Knapp, Karen Stöckler, David Havell, Frédéric Delsuc, Federico Sebastiani, Peter J Lockhart

**Affiliations:** **1**Allan Wilson Centre for Molecular Ecology and Evolution, Institute of Molecular BioSciencesMassey University, Palmerston NorthNew Zealand; **2**Universal College of LearningPalmerston NorthNew Zealand; **3**Institut des Sciences de l'EvolutionUniversité Montpellier IIFrance; **4**Università degli Studi di Firenze, Dipartimento di Biotecnologie Agrarie LaboratoryGenexpress, Polo Scientifico, Sesto FiorentinoItaly; University of California at BerkeleyUnited States of America

## Abstract

*Nothofagus* (southern beech), with an 80-million-year-old fossil record, has become iconic as a plant genus whose ancient Gondwanan relationships reach back into the Cretaceous era. Closely associated with Wegener's theory of “Kontinentaldrift”, *Nothofagus* has been regarded as the “key genus in plant biogeography”. This paradigm has the New Zealand species as passengers on a Moa's Ark that rafted away from other landmasses following the breakup of Gondwana. An alternative explanation for the current transoceanic distribution of species seems almost inconceivable given that *Nothofagus* seeds are generally thought to be poorly suited for dispersal across large distances or oceans. Here we test the Moa's Ark hypothesis using relaxed molecular clock methods in the analysis of a 7.2-kb fragment of the chloroplast genome. Our analyses provide the first unequivocal molecular clock evidence that, whilst some *Nothofagus* transoceanic distributions are consistent with vicariance, trans-Tasman Sea distributions can only be explained by long-distance dispersal. Thus, our analyses support the interpretation of an absence of *Lophozonia* and *Fuscospora* pollen types in the New Zealand Cretaceous fossil record as evidence for Tertiary dispersals of *Nothofagus* to New Zealand. Our findings contradict those from recent cladistic analyses of biogeographic data that have concluded transoceanic *Nothofagus* distributions can only be explained by vicariance events and subsequent extinction. They indicate that the biogeographic history of *Nothofagus* is more complex than envisaged under opposing polarised views expressed in the ongoing controversy over the relevance of dispersal and vicariance for explaining plant biodiversity. They provide motivation and justification for developing more complex hypotheses that seek to explain the origins of Southern Hemisphere biota.

## Abstract 2

A phylogenetic analysis of Nothofagus species provides evidence for their transoceanic dispersal during the Tertiary, and helps resolve the debate about the origins of plant biodiversity in the Southern Hemisphere

## Introduction

**Figure 1:**
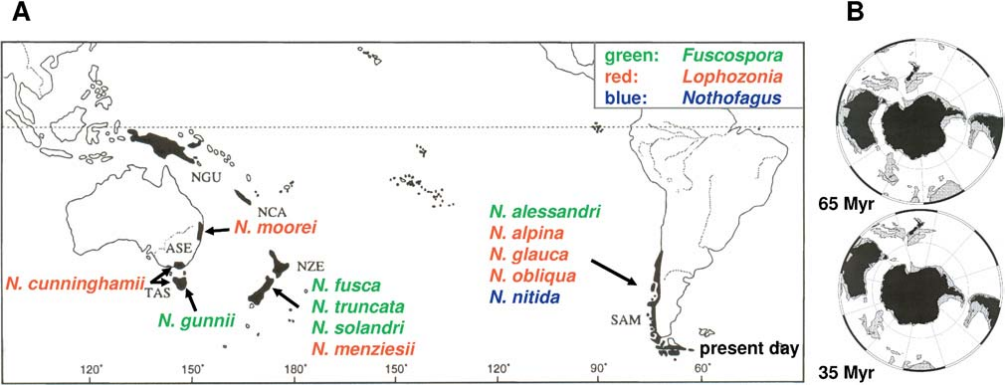
Southern Hemisphere Maps and Present-Day *Nothofagus* Distribution (A) Transoceanic distribution of *Nothofagus* subspecies *Lophozonia* and *Fuscospora* and South American species N. nitida (subgenus *Nothofagus*). Map adapted from Swenson et al. [43]. ASE, Australia; NCA, New Caledonia; NGU, New Guinea; NZE, New Zealand; SAM, South America; TAS, Tasmania. (B) Relationship of Australia, New Zealand, and South America 65 Myr and 35 Myr before present, reconstructed from http://www.odsn.de/ (link “Plate Tectonic Reconstructions”).

An important principle of evolutionary inference is that explanations for the past require an understanding of mechanisms and processes applicable in the present [1]. It is perhaps this sticking point more than any other that has polarised views over the relative importance of vicariance and dispersal for explaining extant plant biodiversity. In 1915, Alfred Wegener put forward a testable hypothesis and mechanism that could explain the transoceanic distribution of animal and plant species. In the 21st century, with many DNA studies now implicating the importance of long-distance dispersal for explaining plant biodiversity [2,3,4,5], it is disconcerting that there is currently a very poor understanding of the mechanisms of transoceanic dispersal (but see [6,7,8,9,10]). Indeed, the inference that the seeds of extant *Nothofagus* species are not suited for dispersal across large distances has played a major role in motivating the hypothesis that transoceanic distributions of *Nothofagus* (Figure 1) can only be explained by vicariance [11,12,13,14,15]. This hypothesis posits that following the Cretaceous breakup of Gondwana, *Nothofagus* rafted and evolved in situ upon different Southern Hemisphere lands. Whilst very attractive, this hypothesis fits somewhat uncomfortably with the findings from analyses of morphological and molecular data. In particular, whilst earlier molecular data have been insufficient for rigorous molecular clock analyses, their interpretation has favoured hypotheses of transoceanic dispersal [16,17,18].

Based on the sequence of Gondwana breakup, a hypothesis of vicariance most parsimoniously predicts that Australian *Nothofagus* species should be most closely related to South American species. This follows since South America and Australia were connected via Antarctica until approximately 35 million years (Myr) ago (Figure 1). In contrast, New Zealand is thought to have separated from Australia 80 Myr ago [19,20]. Thus to explain the close relationship between Australian and New Zealand species by vicariance, it is necessary to argue that extinction of Australian and/or closely related South American species has occurred [12]. Whilst this explanation is ad hoc, the fossil record does provide evidence for numerous *Nothofagus* extinctions in Australia, South America, and New Zealand [21,22,23].

However, the fossil record has also been interpreted as indicating multiple events of transoceanic dispersal of *Nothofagus* from Australia to New Zealand. Whilst the extinct “ancestral” *Nothofagus* pollen type occurred in New Zealand prior to the breakup of Gondwana, *Fuscospora* pollen first appeared in New Zealand during the Palaeocene (65 Myr ago) and *Lophozonia* pollen first appeared during the late Eocene (50 Myr ago; [24]). Sixty-five Myr ago the Tasman Sea had already reached its present-day size [19,20]. Hence it is possible that extant New Zealand *Nothofagus* subgenera did not have the opportunity to reach New Zealand via overland migration. Hill [25] has also described the species *Nothofagus cethanica,* which first appeared in Oligocene macrofossils from Tasmania. This species shares unique features with extant N. fusca and N. truncata from New Zealand and may share a sister relationship with these species explained by trans-Tasman Sea dispersal [26].

A contribution to the debate over the relative importance of vicariance and dispersal can be made by estimating the divergence times of extant species. However, DNA sequences of insufficient length have prevented robust molecular clock analyses from being undertaken. For this reason, we report the sequencing of a 7.2-kb chloroplast genome fragment covering the gene regions (*trnL–trnF* and *atpB–psaI;* see Table 1 for accession numbers) for 11 species of three *Nothofagus* subgenera (*Lophozonia, Fuscospora,* and *Nothofagus*). Our aim has been to date divergence of extant species in the subgenera *Lophozonia* and *Fuscospora.* We have carried out relaxed molecular clock analyses using the methods of Sanderson [27,28] and Thorne et al. [29]. Our findings are that, whilst vicariance is likely to explain some transoceanic relationships amongst *Nothofagus* species, phylogenetic relationships between trans-Tasman species in both *Lophozonia* and *Fuscospora* can only be explained by mid- to late-Tertiary transoceanic dispersal.

**Table 1:**
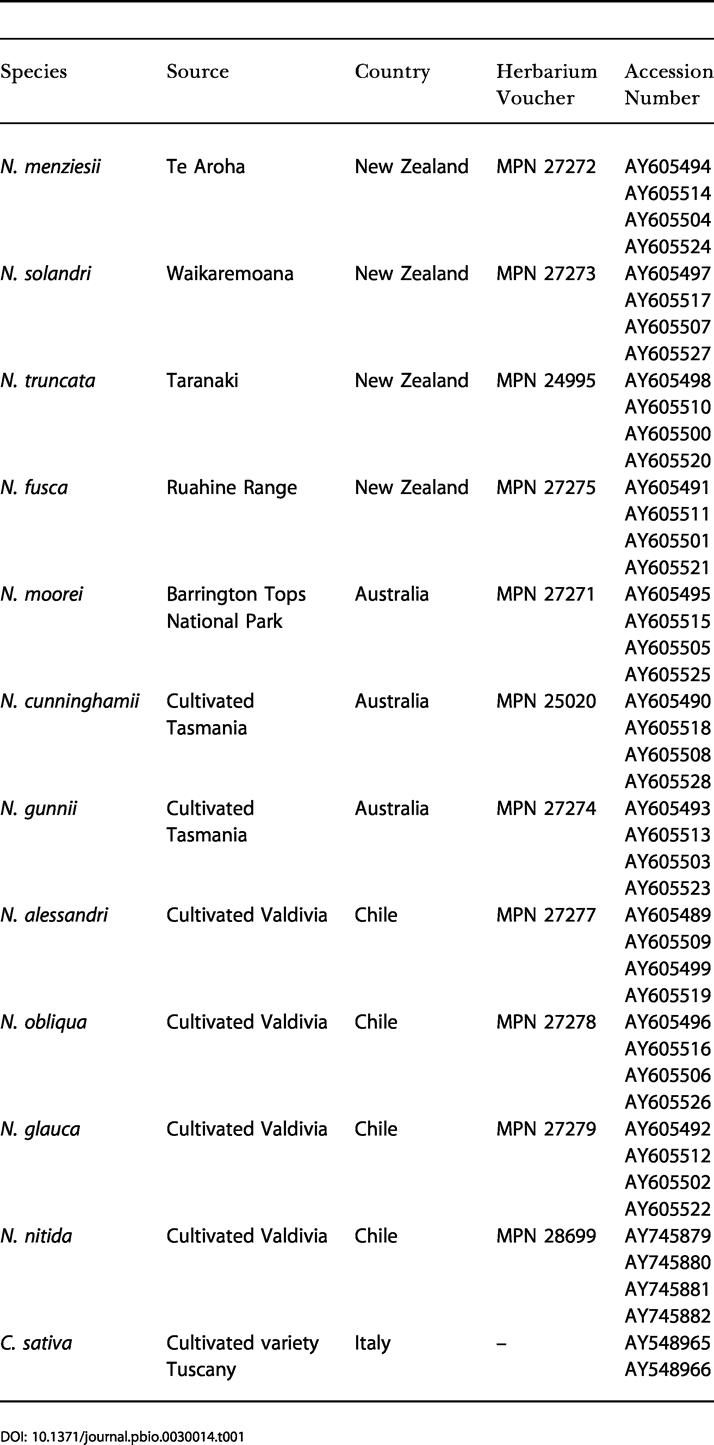
Origin of *Nothofagus* Samples and Sequence Accession Numbers

## Results

**Figure 2:**
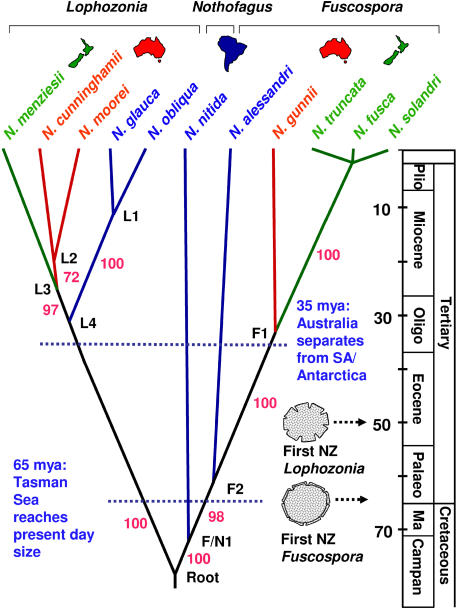
ML Tree Indicating Evolutionary Relationships for *Nothofagus* Species Based on the *atpB–psaI* and *trnL–trnF* Region of the Chloroplast Genome (7,269 bp) Divergence dates (in Myr) were obtained with an F84+ Γ_8_ substitution model using the BRMC approach of Thorne et al. [29]. For the dates indicated, the age of the root node and that of F/N1 were constrained to 70–80 Myr; L2 was also constrained in accordance with fossil data [26] at 20 Myr. Violet numbers show bootstrap values. The pollen grains represent the first appearance of the respective pollen type in the New Zealand fossil record. Plio, Pliocene; Oligo, Oligocene; Palaeo, Palaeocene; Ma, Maastrichian; Campan, Campanian. L1–L4, *Lophozonia* 1–4; F1–F2, *Fuscospora* 1–2; F/N1, *Fuscospora*/*Nothofagus* 1.

Figure 2 shows an optimal maximum-likelihood reconstruction of phylogenetic relationships for chloroplast DNA sequences (7.2-kb comprising the *atpB–psaI* region and the *trnL–trnF* region; 7,269 nucleotide sites) for *Nothofagus* (subgenera or pollen groups (a) *Lophozonia,* (b) *Fuscospora,* and (c) *Nothofagus*) and outgroup Castanea sativa (not shown). In a sensitivity analysis of 60 substitution models, the tree shown in Figure 2 was always recovered with very little difference in branch lengths regardless of the substitution model used. Of all substitution models evaluated, K81uf+G was identified as the best fitting one for the data based on hierarchical likelihood ratio tests and the Akaike Information Criterion. This substitution model and also the F84+ Γ_8_ model were used for further analyses. The latter was included because the Bayesian relaxed molecular clock (BRMC) approach as implemented in the program MULTIDIVTIME (see Materials and Methods) only allows the use of the JC and the F84 models. Thus analysis with the F84+ Γ_8_ model allowed a comparison of date estimates to be obtained using different relaxed molecular clock methods. All nodes of the optimal ML tree recovered in the sensitivity analysis received nonparametric bootstrap support greater than 97%, with the only exception being the grouping of N. cunninghamii with *N. moorei,* which received 72% support.

Divergence times for the nodes in this tree (Figure 2) were estimated using the penalized likelihood (PL) method [27] and BRMC method [29,30,31]. For these analyses, a period of 70–80 Myr was used to calibrate the divergence between the three fossil pollen groups representing subgenera *Lophozonia, Nothofagus,* and *Fuscospora.* These four pollen groups all first appeared in the fossil record approximately 75 Myr ago [32]. A second constraint of a minimum of 20 Myr for the divergence of N. cunninghamii and N. moorei was also used. This constraint was based on observations reported by Hill [26] that 20-Myr-old fossils intermediate between N. moorei and N. cunninghamii were recorded from Tasmania and that fossils closely resembling N. moorei were also present at that time. The inferred ages for the remaining nodes of the tree, obtained under the F84+ Γ_8_ model of substitution are given in Table 2 and graphically illustrated on Figure 2. The variance on these estimates was low and the values were little influenced by the choice of substitution model (Table 3). The robustness of the estimates to calibration error was tested by constraining the divergence of Australian and New Zealand sister taxa to 65 Myr (the time before present when the Tasman Sea reached its present position; thus this date provided us with a lower bound for divergence times of trans-Tasman *Nothofagus* disjunctions that might be explained by vicariance). Constraining these two nodes in this way produced unrealistic age estimates for all basal nodes. For example, using the BRMC method, which additionally required a prior expectation to be specified for the age of the root node (which we specified at 75 Myr—the time of appearance of all four extant pollen types), we estimated a more likely age for the root node at 191 Myr. For the PL approach, which does not require specification of a prior, we estimated the age of the root node at 634 Myr. Other basal nodes in both the *Fuscospora* and *Lophozonia* lineages were also much older than reasonably expected (see Table 2).

**Table 2:**
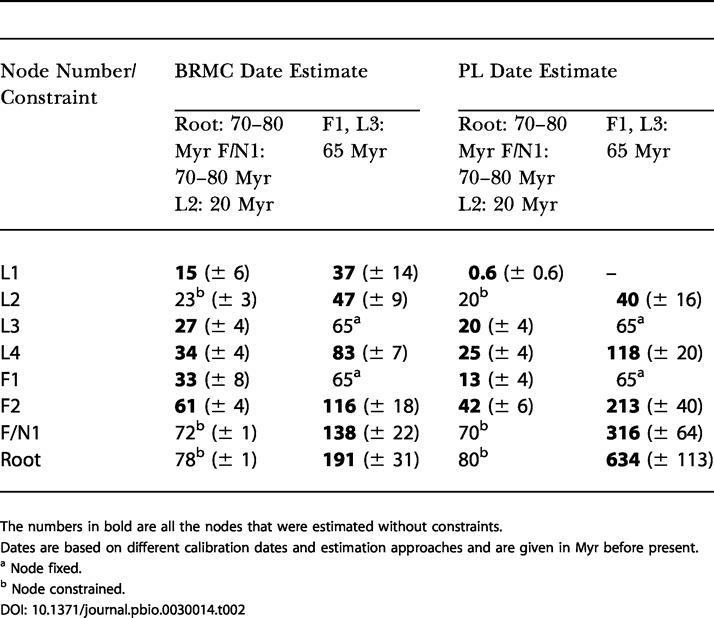
Estimated Divergence Dates and Standard Deviations (in Brackets) of Different *Nothofagus* Clades

The numbers in bold are all the nodes that were estimated without constraints

Dates are based on different calibration dates and estimation approaches and are given in Myr before present

^a^ Node fixed

^b^ Node constrained

DOI: 10.1371/journal.pbio.0030014.t002

**Table 3:**
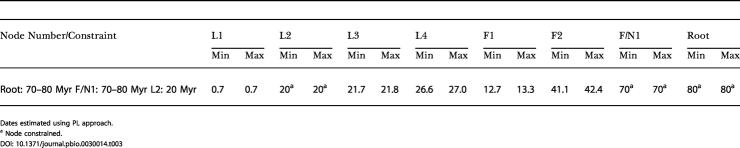
Variation of Estimated Divergence Times (in Myr) under 60 Symmetrical Models of DNA Substitution

Dates estimated using PL approach

^a^ Node constrained

## Discussion

Our findings from molecular clock analyses using five independent calibrations (for four nodes), suggest that the sister relationships of the Australasian (Australia and New Zealand) species within both *Lophozonia* and *Fuscospora* lineages are too young to be explained by continental drift (as indicated by the inferred ages of nodes F1 and L3). Transoceanic dispersal appears the most likely explanation for the trans-Tasman sister relationships indicated in Figures 1 and 2. In contrast, the age inferred for node F2, using both relaxed clock methods is compatible with a hypothesis of continental drift as an explanation for the sister relationship between South American and Australasian *Fuscospora* lineages. The age for node L4, which separates Australasian and South American *Lophozonia,* may also be consistent with vicariance. The BRMC method dates it at 34 Myr before present. However, the PL method estimates this node to be only 25 Myr old, an age too recent to be consistent with vicariance. Thus we regard our results for node L4 as equivocal. Nevertheless, southern beeches are likely to have been present in Antarctica 25 myr ago [33], and thus long-distance dispersal across the young southern ocean between South America and Australia via Antarctica may be conceivable.

The robustness of our phylogenetic inferences has been investigated by varying the substitution model (60 symmetric models were used), estimating the variance of age estimates, and evaluating the influence of calibrations on divergence times. With the exception of the root node, the PL method consistently gave more recent age estimates than did the BRMC method. Both methods showed sensitivity to the number of calibration points used, a finding consistent with recent observations on the performance of relaxed molecular clock methods [34]. In general, the date estimates produced by the BRMC approach were more consistent with the fossil record [26]. A relevant question is whether or not additional calibration points could make date estimates older and thus change our conclusion of trans-Tasman dispersal. We suggest that this may be unlikely, given the observation that constraining a minimum age for trans-Tasman sister species to 65 Myr leads to greatly inflated and unrealistic age estimates for all basal nodes. Hence to explain this finding we would need to invoke a further hypothesis of a dramatic and independent slowing in the rate of evolution in *Lophozonia, Fuscospora,* and *Nothofagus* lineages.

Thus the hypothesis that present-day distribution patterns of *Nothofagus* can be explained by continental drift following the breakup of Gondwana and subsequent extinction of some species [24] can be rejected on the basis of the divergence dates that we have estimated. These dates also indicate that present-day *Nothofagus* species in New Zealand are not the direct descendants of the *Fuscospora* and *Lophozonia* southern beeches that reached New Zealand in the Palaeocene and Eocene eras, respectively [24]. This finding highlights the caution that needs to be taken when interpreting fossil evidence for the apparent first appearance of extant taxa. Fossils that identify specific evolutionary lineages may not necessarily indicate the origins for extant taxa or suggest a continuous presence for these taxa. Similar concerns follow from the findings of molecular analyses for *Ascarina* and *Laurelia* in New Zealand [2,4].

The strength of our molecular analyses highlights the importance of future research into potential mechanisms of long-distance dispersal, and in particular reinvestigation of the transoceanic dispersal properties of *Nothofagus* seeds. For the reasons that we outline in our introduction, it seems likely that only once the mechanisms of long-distance dispersal are understood will hypotheses based on DNA divergence time estimates be truly convincing. DNA sequence analyses have also suggested that long-distance dispersal and continental drift are both important for explaining distributions of the conifer *Agathis* (Araucariaceae) in the South Pacific [35]. Although the molecular evidence for *Agathis* is not as strong as it is for *Nothofagus,* the findings from the molecular studies on these genera highlight the importance of considering more complex hypotheses of relationship in debates concerning the relative importance of dispersal and vicariance.

## Materials and Methods

### 

#### Sequence data

Chloroplast DNA sequences (7.2 kb comprising the *atpB–psaI* region and the *trnL–trnF* region) were determined for each of 11 accessions of *Nothofagus* (subgenera or pollen groups *Lophozonia, Fuscospora,* and *Nothofagus*) sampled in South America, Australia, and New Zealand (see Table 1). These genome regions were also determined for C. sativa (an outgroup taxon from Fagaceae) and aligned using progressive multiple-sequence alignment: ClustalX version 1.81 [36]. This resulted in an unambiguous alignment of 7,269 nucleotide sites. Data are missing for approximately 250 bp of the *atpB* gene and *atpB*–*rbcL* intergene region of *Nothofagus.*

#### Tree building

Phylogenetic analyses were conducted with PAUP* version 4.0b10 [37] under the ML criterion. A model sensitivity test was conducted, investigating a range of 60 symmetrical models of DNA substitution corresponding to the 56 implemented in MODELTEST version 3.06 [38] (http://darwin.uvigo.es/software/modeltest.html) plus F84, F84+I, F84+Γ_8_, and F84+I+Γ_8_ **.** ML parameters of these models were estimated by PAUP* following the approach used in MODELTEST. These parameters were then used to conduct 60 individual ML heuristic searches in PAUP* with tree bisection-reconnection branch swapping and a neighbour-joining starting tree. ML bootstrap proportions were obtained after 100 replications, using the same search strategy and ML parameters as for the analysis of the original dataset.

#### Molecular dating: The PL method

Divergence dates were obtained using the PL method of Sanderson [27] as implemented in the program r8s, version 1.60 [28] (http://ginger.ucdavis.edu/r8s/) with the TN algorithm. The outgroup was excluded using the “prune” command. The degree of autocorrelation within lineages was estimated using cross-validation as suggested by Sanderson [27], and the correcting smoothing parameter λ defined accordingly. Divergence dates were estimated on the 60 ML phylograms recovered in the phylogenetic model sensitivity analysis. Ages for each node across the 60-ML trees were summarized using the “profile” command. Confidence limits on dating estimates were computed by using nonparametric bootstrapping of the original dataset as suggested by Sanderson and Doyle [39]. The program SEQBOOT of the PHYLIP 3.6 package [40] was used to generate 100 bootstrap resampled datasets of 7,269 sites in length. ML branch lengths of the optimal topology were then estimated under the F84+ Γ_8_ model for each of the bootstrap resampled datasets using PAUP*. Divergence estimates were then calculated for each of the 100 bootstrap replicates using r8s to obtain standard deviations on each node by the “profile” command and the settings described above.

#### Molecular dating: The BRMC method

The BRMC approach was applied using the program MULTIDIVTIME as implemented in the Thornian Time Traveller (T3) package [41]. First, the program BASEML of the PAML package version 3.13 [42] (http://abacus.gene.ucl.ac.uk/software/paml.html) was used to estimate the ML parameters of the F84+ Γ_8_ substitution model, using the ML topology previously identified. Second, the program ESTBNEW (ftp://abacus.gene.ucl.ac.uk/pub/T3/) was used to estimate branch lengths of the ML topology and the corresponding variance–covariance matrix. Finally, the program MULTIDIVTIME was used to run a Markov chain Monte Carlo for estimating mean posterior divergence times on nodes with associated standard deviations from the variance–covariance matrix produced by ESTBNEW. The Markov chain was sampled 10,000 times every 100 cycles after a burn-in stage of 100,000 cycles. We used a 75-Myr (SD = 37.5 Myr) prior [32] for the expected number of time units between tip and root and a prior of 200 Myr for the highest possible number of time units between tip and root. Other priors for gamma distribution of the rate at root node and the Brownian motion constant describing the rate variation (i.e., the degree of rate autocorrelation along the descending branches of the tree) were derived from the median branch length. As for the PL method, the outgroup was not included in this analysis.

## Supporting Information

### Accession Numbers

The GenBank (http://www.ncbi.nlm.nih.gov/) accession numbers for the sequences discussed in this paper are given in Table 1.

## Abbreviations

BRMC: Bayesian relaxed molecular clock
ML: maximum likelihood
Myr: million years
PL: penalized likelihood
